# Targeting the EGF Receptor for Ovarian Cancer Therapy

**DOI:** 10.1155/2010/414676

**Published:** 2009-12-28

**Authors:** Reema Zeineldin, Carolyn Y. Muller, M. Sharon Stack, Laurie G. Hudson

**Affiliations:** ^1^Department of Pharmaceutical Sciences, College of Pharmacy, University of New Mexico, Albuquerque, NM 87131-0001, USA; ^2^Department of Obstetrics and Gynecology, School of Medicine, University of New Mexico, Albuquerque, NM 87131-0001, USA; ^3^Department of Pathology and Anatomical Sciences, University of Missouri School of Medicine, Columbia, MO 65212, USA

## Abstract

Ovarian carcinoma is the leading cause of death from gynecologic malignancy in the US. Factors such as the molecular heterogeneity of ovarian tumors and frequent diagnosis at advanced stages hamper effective disease treatment. There is growing emphasis on the identification and development of targeted therapies to disrupt molecular pathways in cancer. The epidermal growth factor (EGF) receptor is one such protein target with potential utility in the management of ovarian cancer. This paper will discuss contributions of EGF receptor activation to ovarian cancer pathogenesis and the status of EGF receptor inhibitors and EGF receptor targeted therapies in ovarian cancer treatment.

## 1. Introduction

Ovarian carcinoma is the leading cause of death from gynecologic malignancy, with an estimated 15 520 deaths in the USA in 2008 [1]. Ovarian cancer is a highly metastatic disease that is rarely detected when disease is confined to the ovary (stage I) and 5-year survival is >90%. The great majority of ovarian cancer patients are initially diagnosed with disseminated intra-abdominal disease (stages III–IV) and have a 5-year survival of <20% [2]. Clinically, ovarian tumors often involve the ovary and omentum, with diffuse, multifocal intraperitoneal metastases and malignant ascites [2, 3]. The combined factors of late diagnosis and the cellular and molecular heterogeneity of ovarian cancers hamper efforts to effectively treat this disease.

For many cancers, including those of the ovary, there is growing emphasis on the identification and development of targeted therapies to disrupt specific molecular pathways contributing to disease progression [4]. The epidermal growth factor (EGF) receptor is one such molecular target. The EGF receptor impinges on multiple key hallmarks of cancer defined by Hanahan and Weinberg [5] and the EGF receptor is associated with a gene expression pattern unique to invasive tumor cells [6]. Aberrant expression and activity of the EGF receptor is generally recognized to have a deleterious impact on the clinical outcome of cancer patients which has fueled development of targeted therapeutics (reviewed in [7–12]). This paper will discuss potential contributions of EGF receptor activation to ovarian cancer pathogenesis and the status of EGF receptor inhibitors and EGF receptor targeted therapies in ovarian cancer treatment.

## 2. The EGF Receptor in Ovarian Cancer

The EGF receptor is a member of the receptor tyrosine kinase (RTK) family of growth factor receptors and the founding member of the ErbB subfamily that includes four proteins: ErbB1 (EGF receptor), ErbB2 (HER-2), ErbB3 (HER-3), and ErbB4 (HER-4). The ErbB receptors are single membrane spanning proteins possessing intrinsic tyrosine kinase catalytic activity. Ligand binding promotes EGF receptor homo- and heterodimerization with ErbB family members, activation of the intracellular tyrosine kinase domain, and stimulation of numerous downstream signaling cascades associated with cell growth and survival, increased angiogenesis, and tumor metastasis (reviewed in [7–10], [13–17]). 

The most common form of ovarian cancer arises from the ovarian surface epithelium (OSE). The OSE expresses EGF receptors in vivo and EGF receptor activity is implicated in gonad development, growth and differentiation of the ovarian follicle, and postovulatory repair [18–20]. It has been proposed that EGF stimulation of the OSE contributes to its rapid post-ovulatory proliferation and to epithelial-mesenchymal transition (EMT) of OSE cells within the ruptured follicle. Malfunctions in post-ovulatory repair are believed to contribute to formation of epithelial inclusion cysts, which are the preferential sites of malignant transformation [15, 21, 22]. The normal OSE responds to EGF receptor generated signals by displaying a phenotypic plasticity characterized by transition between epithelial and fibroblastic phenotypes, a characteristic usually limited to immature, regenerating, or neoplastic epithelia [23]. These attributes of the adult OSE suggest that this tissue is “primed” to respond to the EGF receptor during tumor development and progression. 

In addition to its role in normal ovarian epithelium, there is abundant evidence of aberrant EGF receptor and/or ligand expression in ovarian cancer. A recent review [15] provides an excellent and comprehensive summary of immunohistochemical studies evaluating ErbB receptor and ErbB ligand expression in malignant ovarian tumors. Briefly, published reports estimate EGF receptor expression in 10–70 percent of human epithelial ovarian cancer cases (reviewed in [15]). A smaller subset of studies has examined amplification of the EGF receptor gene in ovarian cancer. An advantage of this approach is the relative stability of DNA in archived samples, but because EGF receptor overexpression can occur in the absence of gene amplification, these studies may underestimate the frequency of elevated EGF receptor protein in tumors. Despite this caveat, EGF receptor gene amplification is detected in ~10–20 percent of ovarian cancer cases [24–26], with low-level gains detected more frequently in 43 percent of tumors [24]. Thus, based on detection of protein or gene amplification, there is strong evidence for elevated EGF receptor expression in a significant fraction of ovarian cancer cases. 

Overall, elevated EGF receptor is associated with less favorable disease outcomes in a number of human tumors [17, 27–29]. Despite evidence for EGF receptor expression in ovarian tumors [15], studies on the relationships between receptor and patient outcomes do not provide a uniform picture on the clinical consequences of elevated EGF receptor levels. Based on studies with normal tissue reference controls, elevated EGF receptor levels significantly correlated with aggressive disease characteristics [24] and high tumor EGF receptor expression was proposed as the most significant prognostic factor for disease-free and overall survival [30]. An overall conclusion that aberrant EGF receptor status is a factor in ovarian cancer outcome is supported by a meta-analysis study revealing a relationship between EGF receptor and decreased survival [31], and the abundant evidence linking EGF receptor to poor patient outcome in other cancers of epithelial origin.

## 3. Consequences of EGF Receptor Activation in Ovarian Cancer

A limited number of studies examine activated (tyrosine phosphorylated) EGF receptor in ovarian tumors and overall, little attention has been given to receptor activation status and disease parameters. In one study, 11.8 percent of ovarian tumors were positive for phosphorylated EGF receptor (pEGFR) but no clinicopathological parameter or survival differences were noted [32]. In another study, twenty-four heavily pretreated patients with epithelial ovarian cancer all had detectable EGF receptor and p-EGFR (Y1148), suggesting that EGF receptor activation might be more evident in advanced disease [33]. We conducted a tumor tissue array analysis and found evidence for pEGFR in approximately 1/3 of ovarian tumor samples [34]. EGF receptor activation was statistically positively correlated with matrix metalloproteinase (MMP)-9 expression, a protein associated with tumor invasion and metastasis. Together, these in vivo data indicate that activated EGF receptor is present in ovarian tumor specimens, likely driving aspects of tumor behavior.

The mitogenic effects of EGF receptor activation in ovarian tumor cells are well documented. EGF increases the growth potential of primary ovarian surface epithelial (OSE) cells in culture [35] and gene expression profiling of normal rat ovarian surface epithelium following EGF treatment demonstrates EGF-dependent activation of genes involved in cell cycle and proliferation, apoptosis, and protein turnover [36]. In addition, malignant transformation of rat OSE cells results in alteration of downstream effectors of the EGF receptor pathway [36]. Regarding ovarian tumor cells, numerous studies demonstrate that autocrine and paracrine stimulation of the EGF receptor promotes ovarian tumor cell growth (reviewed in [37, 38]). Furthermore, blockade of EGF receptor activation or signaling inhibits ovarian tumor cell growth in vitro and in vivo (reviewed in [37]).

In addition to fostering cell growth, activation of the EGF receptor is associated with stimulation of metastasis-associated cellular responses. Many aspects of tumor metastasis resemble features of epithelial-mesenchymal transition (EMT) [39–43]. Notably, EGF receptor activation is capable of driving EMT-associated events in epithelial ovarian carcinoma cells in culture including migration and invasion, disruption of E-cadherin-mediated intercellular junctions, and production of matrix degrading proteinases (reviewed in [37, 38, 44, 45])**. ** In contrast to the well-defined events that characterize EMT in development, tumor-associated EMT is currently viewed as a continuum of phenotypic plasticity and gain of mesenchymal characteristics. Tumor phenotype likely reflects the particular complement of EMT regulatory factors expressed in cells or within the tumor microenvironment [42–45]. The functional consequences of this phenotypic plasticity are not fully understood, but may play a role in modulation of cell survival in suspension (ascites), chemoresistance, and intraperitoneal anchoring of metastatic lesions (reviewed in [42, 44, 46]). 

Based on the evidence that (1) ovarian tumors share certain characteristics (EGF receptor overexpression and activation) with tumors approved for treatment with EGF receptor inhibitors, (2) receptor activation drives tumor-relevant responses in ovarian tumor cells, and (3) ovarian tumor growth is reduced by EGF receptor directed therapeutics in preclinical models, the EGF receptor inhibitors have moved forward into clinical trials for ovarian cancer and are discussed in the following section.

## 4. Clinical Status of EGF Receptor Inhibition in Ovarian Cancer

With the advent of better understanding of the molecular mechanisms contributing to ovarian cancer, novel receptor targeted therapeutics or “biologic therapeutics” either administered alone or in combination with conventional chemotherapy have become a rapidly developing strategy in clinical trials design. Based on expression of the EGF receptor in ovarian cancer and the known consequences of receptor activation, this pathway could be a prime target for therapeutic blockade [4]. Numerous anti-EGF receptor agents are under active development and each compound has subtle differences in target binding, downstream signaling, ease of administration and toxicity profiles. Yet despite favorable preclinical studies using EGF receptor antagonists, clinical trial outcomes in ovarian cancer have been overall disappointing. Investigations are underway to understand the mechanism of escape from EGF receptor blockade as well as to identify clinical predictors of antagonist response. The following sections will summarize the success and shortcomings of these agents in ovarian cancer trials. 

The majority of EGF receptor inhibitor agents in clinical trial development fall into two categories: small molecule tyrosine kinase inhibitors (TKIs) that compete with ATP for its binding site in the tyrosine kinase domain or monoclonal antibodies (MAbs) against the extracellular domain that interfere with ligand binding and/or receptor dimerization. Additional EGF receptor directed therapeutic strategies include development of EGF vaccines, receptor downregulation by antisense oligonucleotides [47]. EGF receptor dependent targeting of imaging agents, chemotherapeutic agents, and toxins will be discussed later in this paper. 

A significant clinical difference between the small molecule TKIs and MAbs is that the TKIs are orally administered and require daily dosing (especially the reversible inhibitors) to maintain target blockade whereas the MAbs are given intraveneously usually weekly or every 2 weeks. The TKIs and MAbs share a toxicity profile which includes fatigue, diarrhea, and a robust acneiform rash. The cutaneous rash has been described as a clinical indicator of EGF receptor blockade due to abrogation of receptor signaling in nontumor tissues such as the skin and gut mucosa [47]. In addition, hypersensitivity reactions are a concern with MAbs, especially the nonhumanized or chimeric agents. Several TKIs and MAbs are FDA approved for treatment of specific solid tumors, yet none have performed well enough in ovarian cancer trials to warrant such approval (Table 1). Additional compounds are under clinical development in ovarian cancer and other solid tumors (Table 2).

### 4.1. EGF Receptor Specific Inhibitors

In clinical trials EGF receptor inhibitors have been administered as single agents and in combination with chemotherapy. Generally the trials are conducted in patients with recurrent ovarian cancer, and often patients have been heavily pretreated before receiving the targeted therapeutics. The common dosing schedules from multiple Phase I trials for the oral TKIs are shown in Table 1. Gefitinib alone (500 mg) performed poorly in Phase II trials with minimal clinical response for ovarian cancer patients. The only responder had an activating mutation in the EGF receptor catalytic domain similar to the mutations evident in responsive lung cancer patients [48]. Erlotinib alone (150 mg) performed slightly better with 6% of the patients responding based on tumor regression and 44% of patients had stable disease [4]. Gefitinib has been combined with cytotoxic chemotherapy such as carboplatin, paclitaxel, topotecan, oxaliplatin, vinorelbine, and the aromatase inhibitor anastrazole in multiple Phases I and II trials with some patients responding to treatment [4, 47]. Eroltinib has been combined with carboplatin, docetaxel, paclitaxel, and the VEGFR inhibitor bevacizumab [4]. Several of these trials were performed as front line treatment after cytoreductive surgery demonstrating good clinical and some pathologic complete response rates, but the response rates do not appear dramatically different when compared to historic controls for conventional therapy alone. The pipeline of EGF receptor tyrosine kinase inhibitors continues to expand (Table 2). A randomized Phase II trial of the irreversible EGF receptor inhibitor CI-1033 was performed in a heavily pretreated population of women with recurrent ovarian cancer. Two different oral dose regimens were given (50 mg versus 200 mg daily) for 21 days. Unfortunately there were no responders to single agent treatment and no association between baseline ErbB expression and disease stability [49]. Future studies will likely see these new agents in combination with cytotoxic and other biologic agents.

There are many possible reasons to account for the modest responses to EGF receptor inhibitors. The oral tyrosine kinase inhibitors can be difficult to use in this patient population, as advanced disease causes loss of bowel function and potential unreliable absorption of drug. Another significant concern is the lack of validated biomarkers for response to these TKIs. To date, activating mutations in the EGF receptor kinase domain are the only known predictors of response, but these mutations have not been fully explored in ovarian tumors.

The monoclonal antibodies against the EGF receptor ligand binding domain have some pharmacologic advantages and may perhaps lead to better clinical outcomes compared to the TKIs. Cetuximab is the prototype MAb and has been administered alone or in combination with carboplatin +/− paclitaxel. A Gynecologic Oncology Group (GOG) Phase II trial of cetuximab and carboplatin in platinum sensitive recurrent ovarian cancer showed a 35% response rate (partial and complete responses) in patients with tumors displaying EGF receptor overexpression documented by immunohistochemistry (IHC). Of note, 93% of patients had overexpression of EGF receptor in the primary archived tumor as determined by immunohistochemistry [50]. Although it is tempting to conclude that EGF receptor immunohistochemical analysis of formalin fixed, paraffin embedded tissue is of predictive value for response rate, this has been neither quantified nor validated. A Phase II trial of EMD 72000 (matuzumab) given at 800 mg IV weekly enrolled 37 women with heavily pretreated platinum resistant recurrent ovarian cancer. EGF receptor status was not evaluated for entry criteria or for correlation to clinical response and there were no objective responses in this group when matuzumab was used as monotherapy [51]. The authors concluded that matuzumab monotherapy was not effective for this heavily pretreated group of women. Panitumumab is a fully humanized EGFR MAb under active investigation, particularly in lung and colorectal cancer. It is expected to elicit fewer hypersensitivity reactions than the chimeric human/mouse cetuximab, but to date, there is little direct clinical trial emphasis in ovarian cancer.

### 4.2. Dual Receptor Inhibition

Dual inhibition of ErbB receptor family members is an interesting approach for targeted therapy as much of the signaling is generated by heterodimers, particularly heterodimers of EGF receptor and ErbB2. Lapatinib is an oral small molecule tyrosine kinase inhibitor that reversibly inhibits both ErbB1 and ErbB2. It is well tolerated alone and in combination with chemotherapy as determined by Phase I trials [4, 47]. Our group recently completed a Phase I/II trial of weekly metronomic carboplatin and paclitaxel in combination with lapatinib (1250 mg daily) in 25 evaluable patients with recurrent ovarian cancer. Interval evaluation showed a 50% response rate (complete and partial response) with the expected gastrointestinal and hematologic toxicities [52]. The final analysis and publication of this study is pending. Canertinib (CI-1033) is a newer oral dual TKI which inhibits autophosphorylation of all ErbB receptors including a highly tumorigenic, constitutively active mutant form of the EGF receptor (EGFRvIII) [47]. This agent showed no significant activity as a single agent in a Phase II study in patients with recurrent ovarian cancer. 

Monoclonal antibody dimerization inhibitors have shown the most promise in preclinical studies. Pertuzumab is the prototype of this inhibitor class and prevents ErbB2/HER2 dimerization with the EGF receptor, ErbB3/HER3, and ErbB4/HER4 leading to inhibition of MAP kinase and PI3 kinase signaling. A Phase II trial was conducted by Gordon et al. that included 123 patients with recurrent ovarian cancer (the majority platinum resistant). Two different dosing strategies of pertuzumab as a single agent demonstrated an overall response rate of 4.3% and a mean response duration of 18.6 weeks [53]. Only 28 patients had biopsy material accessible for evaluation of phosphorylated HER2 (pHER2) status by ELISA. Of this group only 8 patients had pHER2 + tissues with one patient in this group experiencing a partial response. The 20 other tumors did not show pHER2 expression and there were no treatment responses in this group [53]. This suggests that pHER2 rather than HER2 overexpression may be a viable biomarker for response although validation studies are desperately needed. Two ongoing randomized Phase II trials in relapsed ovarian cancer are evaluating pertuzumab versus placebo in combination with gemcitabine or carboplatin [54, 55]. In these trials treatments were tolerated, but clinical response endpoints have not yet been reached. In an early analysis of the data, low ErbB3/HER3 mRNA levels as measured in 122 of the 130 patient archival tumor tissues appeared to predict clinical benefit in the cohort receiving gemcitabine + pertuzumab versus the gemcitabine + placebo group [54]. Final analyses of both pertuzamab trials are pending. Additional monoclonal antibodies developed to inhibit EGF receptor family members are listed in Table 2 and studies to test the toxicity and efficacy of these agents in ovarian cancer are needed.

## 5. EGF Receptor as a Targeting Molecule for Imaging Agents and Therapeutics

In addition to therapies directed against the EGF receptor as discussed previously, this receptor has been used to deliver imaging agents or therapeutics to tumors. To target the EGF receptor on tumor cells, EGF receptor ligands or anti-EGF receptor MAbs are incorporated into complexes containing a therapeutic or imaging agent. EGF receptor ligands such as mouse EGF can be conjugated through its N-terminus without affecting receptor binding ability. In contrast, human EGF has two additional amino groups due to internal lysines, and their conjugation can interfere with receptor binding [56]. For that reason mouse EGF rather than human EGF is usually employed for EGF receptor targeting. Novel peptides that specifically bind to EGF receptor provide alternative targeting moieties. Such peptides have been identified either through screening of a virtual peptide library [57], or through screening phage display libraries [58] for peptides that specifically bind to the EGF receptor, including lysine-deficient EGF variants [56]. EGF receptor-targeting moieties are conjugated with imaging or therapeutic agents such as radionuclides, cancer chemotherapeutic agents, toxins, RNase, or photosenstizers. In addition, delivery of oligonucleotides or expression vectors to either suppress or express certain genes in EGF receptor-positive cells through the use of viral or nonviral delivery systems has been reported. Recently more complex systems have been designed that employ various nanocarriers as targeted delivery systems. 

The simplest form of an EGF receptor-targeting complex is radiolabelled-EGF, TKI inhibitor, anti-EGF receptor MAb, or engineered anti-EGF receptor fragments, which can be used for in vivo imaging or for therapeutic purposes [59, 60]. The targeted radionuclide delivery serves as a cytotoxic agent by itself and has been employed in boron neutron capture therapy [61, 62], although optimal therapeutic effects may not be achieved with stand alone boron therapy [63]. Radionuclides as imaging agents can be used to evaluate whether tumors are EGF receptor positive and thus likely to respond to EGF receptor-targeted therapies, or monitor response to therapy. Imaging techniques used to detect EGF receptor-expressing tumors in small animals include positron emission tomography (PET), magnetic resonance (MR), and single photon emission computed tomography (SPECT) [59, 60, 64]. These techniques involve positron emitting radionuclides (such as ^11^C, ^18^F, among others), beta emitters (such as Technetium (^99m^Tc) and Lutetium (^177^Lu)), gamma emitters (such as iodide (^125^I) and Indium (^111^In)), and alpha emitters (such as astatine (^211^At) and bismuth (^212^Bi, ^213^Bi)) [59, 60, 64, 65]. Numerous preclinical studies indicate that tumor targeting can be achieved through the EGF receptor; however, most of these studies did not include ovarian tumor models. 

In addition to radionuclides, cancer chemotherapeutic agents such as cisplatin [66], doxorubicin [67, 68], carminomycin [69], and tyrosine kinase inhibitors [70, 71] have been delivered to EGF receptor-positive cells through conjugation to EGF or to anti-EGF receptor mAb either directly or through a polymer linker. Numerous toxin conjugates that inhibit specific molecular targets within the cell have been delivered to EGF receptor-positive cells including pseudomonas exotoxin (PE) [72], amanitin [73], gelonin [74], and ricin chain A [75–78]. Furthermore, RNases targeted to the EGF receptor were cytotoxic to cancer cells [79–83] and photosensitizers used for photodynamic therapy have been successfully targeted to EGF receptor-positive cells [84–87]. Phase I clinical trials for TP-38 which is a fusion of a mutated PE and the EGF receptor ligand transforming growth alpha demonstrate that it is well tolerated with promising clinical response in patients with recurring malignant brain tumors [88]. The main challenges to expanding use of these toxin conjugates in clinical trials include reducing their immunogenicity by shielding the toxin portion of the complex, and the need to improve delivery to solid tumors [72].

EGF receptor targeted approaches have been used for viral and nonviral gene delivery to cells. As an example of viral systems, avidin-adenovirus (ADV) that expresses GFP was functionalized with EGF, and GFP expression was enhanced in EGF receptor-overexpressing cells compared to cells that moderately express EGF receptor or relative to naked or PEG-ADV [89]. DNA/polycation complexes have been employed for efficient gene delivery as nonviral systems. EGF or anti-EGF receptor MAb was conjugated to cationic polymers such as poly-L-lysine (PLL) [90–95] or polyethyleneimine (PEI) [96–102] that are positively charged and thus interact with negatively charged oligonucleotides or expression vectors. These systems efficiently transfected tumor cells in a receptor-dependent fashion. A number of strategies to improve EGF receptor-specific gene transfer or specificity include PEG or poly–L-glutamic acid (PLG). Other modifications that enhance EGF receptor gene transfer include incorporation of melittin, a membrane active peptide [103], or incorporation of PEG to reduce albumin-caused aggregation [104] and protect the complexes from serum proteins [105]. 

New generations of nanocarriers are under intense investigation as they offer advantages over administering a drug alone or in a simple conjugated targeting moiety. Nanocarriers have numerous benefits including their ability to deliver hydrophobic drugs, increased drug loading, the potential to load multiple drugs or imaging agents, and the ability to functionalize nanocarriers with multiple molecules. Moreover, because of their size these nanocarriers can passively target tumors through the enhanced retention effect caused by large gaps between vascular endothelial cells tissue and defective lymphatic drainage in tumor tissue [106]. In addition to passive targeting, active targeting of cancer tissue can be achieved using nanocarriers functionalized with a targeting moiety such as an EGF receptor ligand or an anti-EGF receptor MAb. Several nanocarriers have been employed as delivery vehicles for drugs or imaging agents to target EGF receptor-positive cancers including liposomes [107–112], gelatin nanoparticles [113, 114], gold [115], dendrimers [116], and carbon nanotubes [117]. These nanocarriers specifically bound to and were internalized by EGF receptor-expressing cancer cells in vitro [109, 115, 116, 118], or preferentially accumulated at tumor sites in vivo [107, 109, 113].

We successfully targeted carbon nanotubes functionalized with EGF and a PEG-fluorescein conjugate to ovarian tumor cells [118]. Specific EGF receptor targeting and cellular uptake was achieved by coating the nanotubes with PL-PEG2000. Furthermore, we find that these vehicles were trafficked to lysosomes, consistent with the fate of ligand-activated EGF receptor (Zeineldin, unpublished data). Lysosomes provide an acidic environment that is conducive to release of drugs attached to the delivery vehicle through acid-labile linkers. This property may allow for the design of therapeutics that will release drugs intracellularly following EGF receptor targeted internalization. In addition, nanocarriers are being developed as efficient drug delivery systems to improve the cellular uptake of certain therapeutic agents such as inhibitory RNA or to enhance the therapeutic efficacy of drugs [106]. A pioneering example of a targeted nanocarrier that just completed phase I trials is CALAA-01. CALAA-01 is a stabilized cyclodextrin-containing polymer that delivers inhibitory RNA through transferrin targeting (Calando Pharmaceuticals: http://www.insertt.com). It is expected that nanotechnology will lead to innovative platforms for targeted drug delivery in future therapeutics.

## 6. Summary and Future Perspectives

There is abundant evidence that EGF receptor activation drives cellular processes linked to ovarian tumor development, tumor cell survival, and metastasis. However, the overall clinical impact of targeting the EGF receptor and its dimers in ovarian cancer, either by monoclonal antibodies or inhibition of the tyrosine kinase domain, has been modest in unselected women with advanced or recurrent ovarian cancer. Although the EGF receptor is a genetically validated target for non-small-cell lung cancer, therapeutic EGF receptor inhibition results in significant tumor regression in only 10–20% of patients [121]. One key goal in applying these agents to ovarian and other cancers will be to identify patients most likely to benefit from targeted therapies and to validate biomarkers of response [2, 4]. This type of preselection is standard in breast cancer, for example, where the estrogen receptor status of a tumor plays a major role in therapeutic decision-making strategy. 

Clearly, a better understanding of in vivo efficacy, improved predictive biomarkers of response, and an understanding of the molecular “escape” pathways for EGF receptor antagonists is needed in ovarian cancer. Given concurrent activation of signaling pathways and pathway crosstalk in tumor cells, inhibition of multiple pathways has been proposed as a strategy to improve the impact of targeted therapeutics [2]. Accordingly, the latest approach in clinical trials is to combine the EGF receptor antagonists with inhibitors of other related or downstream signaling pathways. Phase I clinical trials in solid tumors have been presented recently at the 2009 American Society of Clinical Oncology (ASCO) meeting demonstrating this strategy (Table 3). Agents such as the mTOR inhibitor everolimus and vascular endothelial growth factor receptor inhibitor bevicizumab have been combined with panitumumab, and cetuximab has been combined with the BCR/ABL and src tyrosine kinase inhibitor. Dose limiting toxicities are similar as seen in other combined trials. The impact on biologic endpoints in vivo will be critical to assess the mechanisms of action of these combined therapies. 

Ongoing research continues to identify new and more effective inhibitors of EGF receptor activity, and novel approaches to target antitumor therapies via the EGF receptor. Exploiting the EGF receptor to target and deliver drugs or imaging agents to tumor cells shows promise in preclinical models and an EGF receptor targeted toxin is in clinical trials for glioblastoma [88]. There is resurgence of interest in this strategy based on new generations of nanocarriers with improved drug delivery characteristics and the potential to deliver multiple drugs to tumor cells. Although application of EGF receptor antagonists and EGF receptor targeted therapies to ovarian cancer treatment lags behind that of certain other tumors such as lung and colorectal cancers, lessons learned in using these agents in other diseases are likely to benefit ovarian cancer patients in the future.

## Figures and Tables

**Table 1:** FDA approved EGF receptor inhibitors.

Generic, brand name	Type	Mechanism	Clinical Dose Range (route)	Approved Tumors	Company
Gefitinib, IressaZD1839	Small molecule TKI	Inhibits intracellular EGFR tyrosine kinase phosphorylation	250 mg daily (oral)	Platinum and taxane resistant nonsmall cell lung cancer	Astra-Zeneca

Erlotinib, TarcevaOS-774CP-358774	Small molecule TKI	Inhibits intracellular EGFR tyrosine kinase phosphorylation	100 mg–150 mg daily (oral)	Nonsmall cell lung cancer, pancreatic cancer	OSI Pharmaceuticals/Genentech

Lapatinib, TYKERBGW 572016	Small molecule dual TKI, EGFR-1 and EGFR-2,	Inhibits heterodimerization and her1/her2 phosphorylation	1250 mg daily days 1–21 (oral)	Her2 + breast cancer refractory to herceptin and chemo	Glaxo-Smith Kline

Cetuximab, ErbituxIMC-C225	Human/mouse chimeric MAb	Extracellular domain binding and ligand blockade	400 mg/m^2^ load then 250 mg/m^2^ weekly (IV)	Metastatic colorectal cancer, head, and neck	ImClone

Panitumamab, VectibixABX-EGF	Humanized MAb	Extracellular domain binding and ligand blockade	6 mg/kg every 14 days (IV)	Metastatic refractory colorectal cancer	Amgen/Abgenix

**Table 2:** Non-FDA approved EGFR inhibitors. Data derived from the NCI Drug Dictionary and Clinical Trials Search http://www.nci.nih.gov/Templates/drugdictionary and [4, 47].

Generic or research name	Type	Mechanism	Clinical trial-ovarian cancer, other	Clinical dose range (route)	Company
CI-1033PD 183805Canertinib	Small molecule TKI	Irreversible binding to ATP-binding site EGFR 1, 2, 3, 4	Phase II	50 mg–200 mg daily day 1–21 (oral)	Pfizer

EKB-569Pelitinib	Small molecule TKI	Irreversible binding to TK domain of EGFR 1, 2, 4	None, Phase I in solid tumors	25 mg daily (oral)	Wyeth-Ayerst

PKI-166	Small molecule TKI	Reversible binding to TKI domain EGFR 1, 2	None, Phase I in solid tumors	600 mg–700 mg 2 weeks on/off	Novartis

AV-412	Second generation dual TKI	Reversible binding to TKI domain EGFR 1,2	None, active Phase I trial in solid tumors	Dose escalation daily, dose escalation three times/wk	AVEO Pharmaceuticals

BIBW-2992Tovok	Second generation dual TKI	Irreversible binding to TKI domain EGFR 1, 2	None, Phase I in solid tumors and Phase II in lung, breast, cancer	50 mg daily (oral), 70 mg daily 2weeks on/off	Boehringer Ingelheim's

CUDC-101	Small molecule TKI	Multi-targeted HDAC/EGFR 1, 2	None, Phase I solid tumors	Dose escalation, unknown starting dose	Curis, Inc.

BMS-690154	Small molecule TKI	Binds tyrosine kinase domains of EFGR1, 2 and VEGFR-2	None, Phase I in combo with paclitaxel and carboplatin	Dose escalation, unknown starting dose	Bristol-Myers Squibb

Matuzumab, EMD 72000	Humanized MAb	Extracellular domain binding and ligand blockade	Phase II EGFR+, other head+neck, lung, gastric	800 mg weekly (IV)	EMD Serono/Merk KGaA

Pertuzumab	Humanized MAb	Extracellular her2 ligand blockade, prevents dimers with EGFR-1	Phase II, lung, breast, prostate	840 mg load followed by 420 mg every 3 weeks (IV)	Merck Serono

RO5083945	Glycoengineered MAb	Binds to EGFR extracellular domain, inhibits dimers	None, Phase I EGFR+ solid tumors	Dose escalation start at 50 mg (IV)	Roche Pharmaceuticals

**Table 3:** Clinical trials combining the EGF receptor antagonists with other signaling pathway inhibitors.

American Society of Clinical Oncology (ASCO) 2009 Annual Meeting Proceedings
Phase I trial of bevacizumab + everolimus + panitumumab in refractory solid tumors [117]
Phase I trial of cetuximab and erlotinib in solid tumors [119]
Phase I trial of dasatinib + cetuximab in advanced solid tumors [120]
